# Supplementary material for: Elevated Ozone Concentration and Nitrogen Addition Increase Poplar Rust Severity by Shifting the Phyllosphere Microbial Community
Source: J Fungi (Basel). 2022 May 18;8(5):523. doi: 10.3390/jof8050523 (PMC9148057; doi:10.3390/jof8050523)
Supplement: Supplementary file 1 [file jof-08-00523-s001.zip › Supplementary files/Figure S2.pdf]

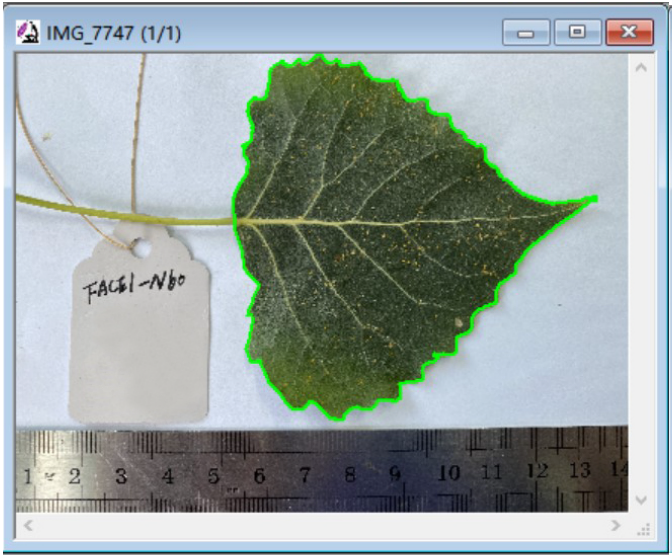

1. Spatial calibration and outline along the edge of the leaf.

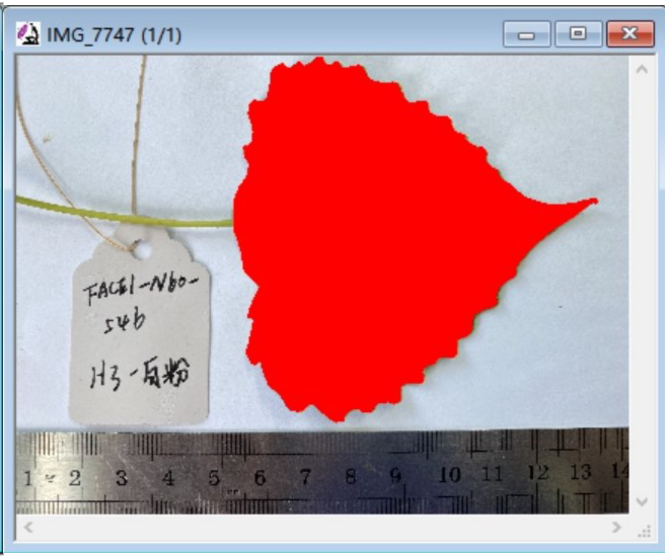

2. Measure the area of selected leaf.

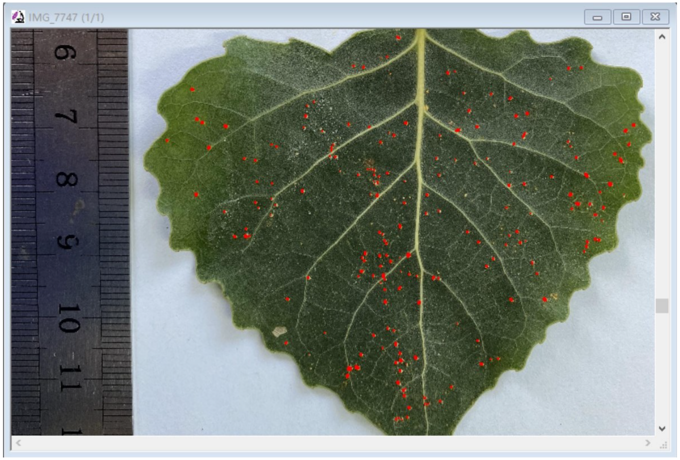

3. Use color separation method to select uredinia.

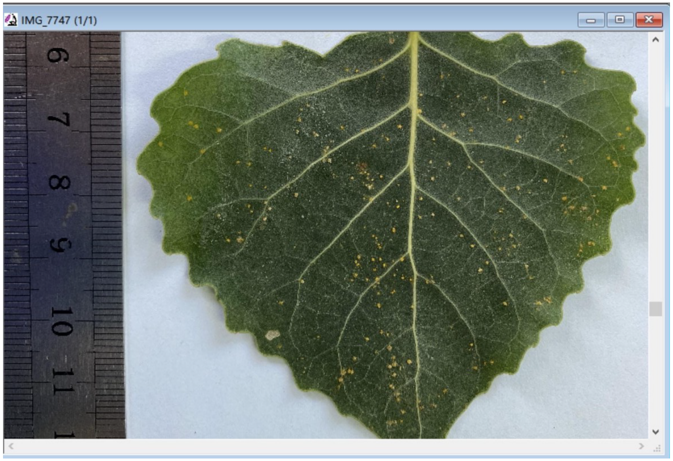

4. Artificially adjust the uredinia numbers compared to images to avoid mistaking.
