# Supplementary figures and images for: Elevated Ozone Concentration and Nitrogen Addition Increase Poplar Rust Severity by Shifting the Phyllosphere Microbial Community
Source: J Fungi (Basel). 2022 May 18;8(5):523. doi: 10.3390/jof8050523 (PMC9148057; doi:10.3390/jof8050523)

A-O<sub>3</sub>

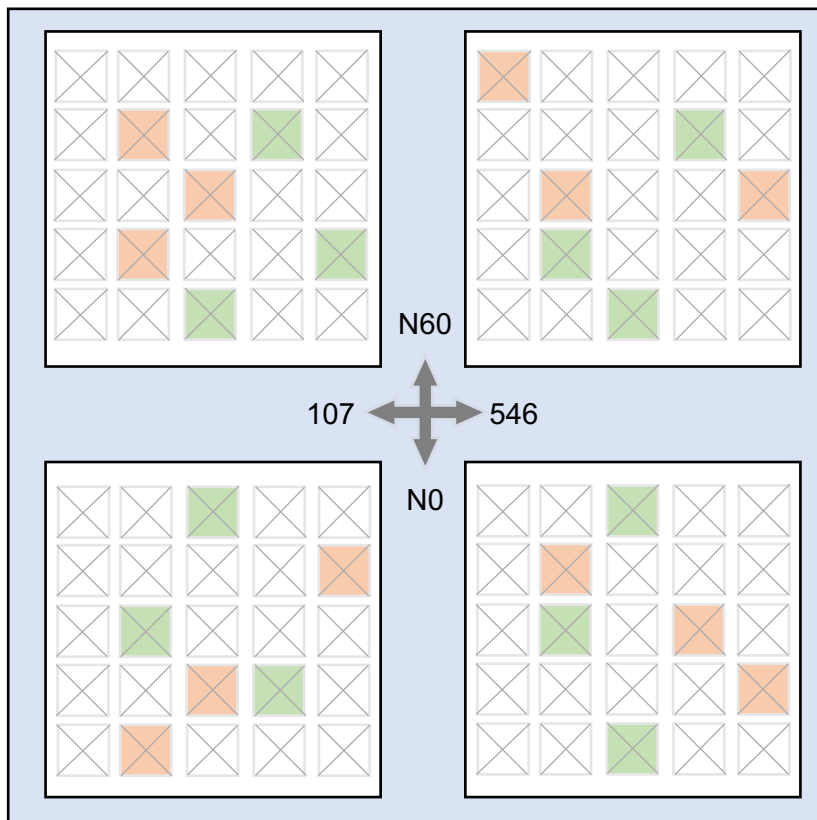

E-O<sub>3</sub>

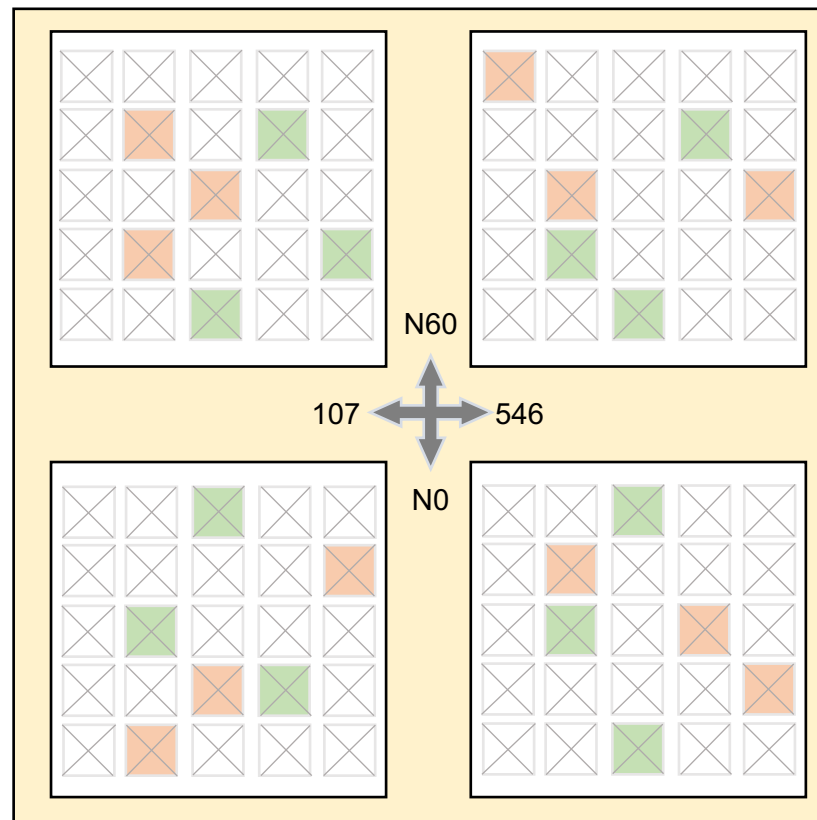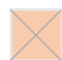

Rust-infected poplars

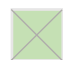

Non-infected poplars

Supplement: Supplementary file 1 [file jof-08-00523-s001.zip › Supplementary files/Figure S1.pdf]

## Bacteria

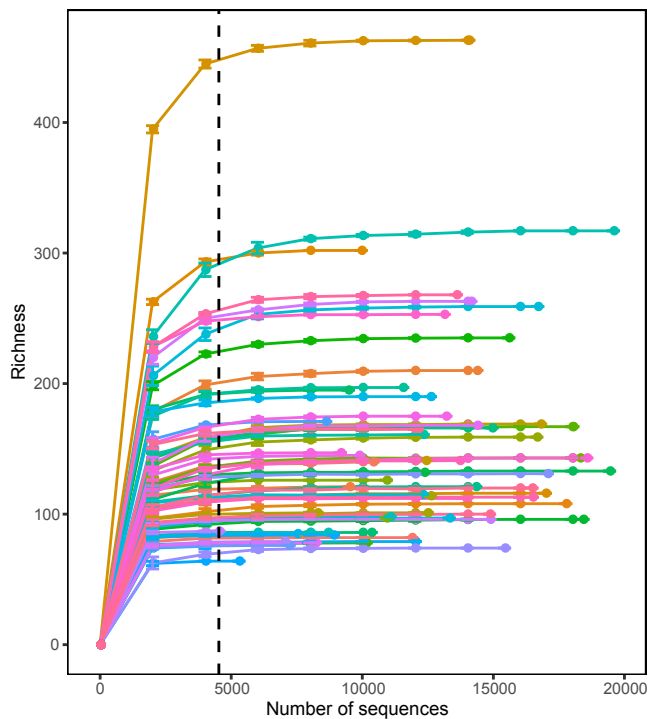

## Fungi

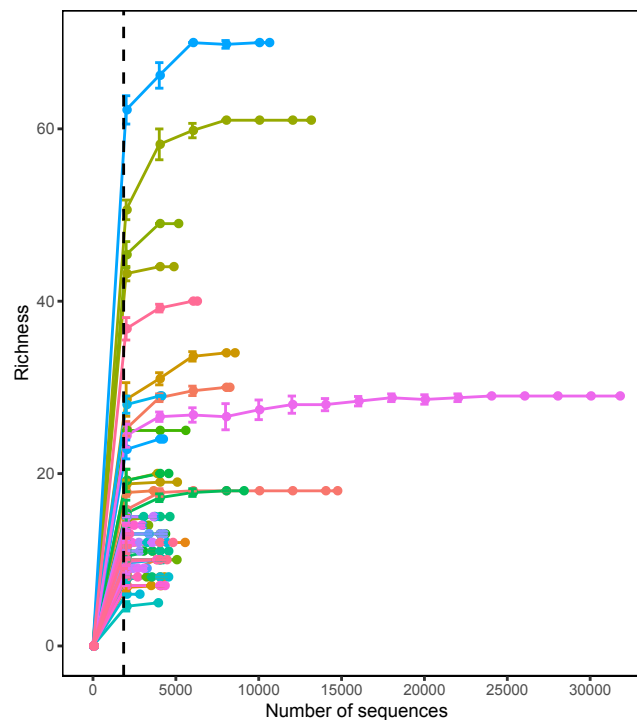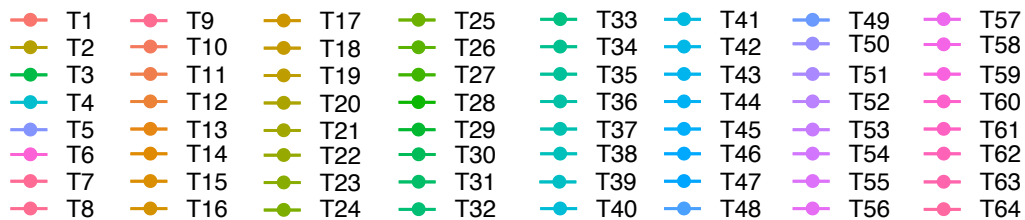

Supplement: Supplementary file 1 [file jof-08-00523-s001.zip › Supplementary files/Figure S3.pdf]

**a** Bacteria beta-diversity

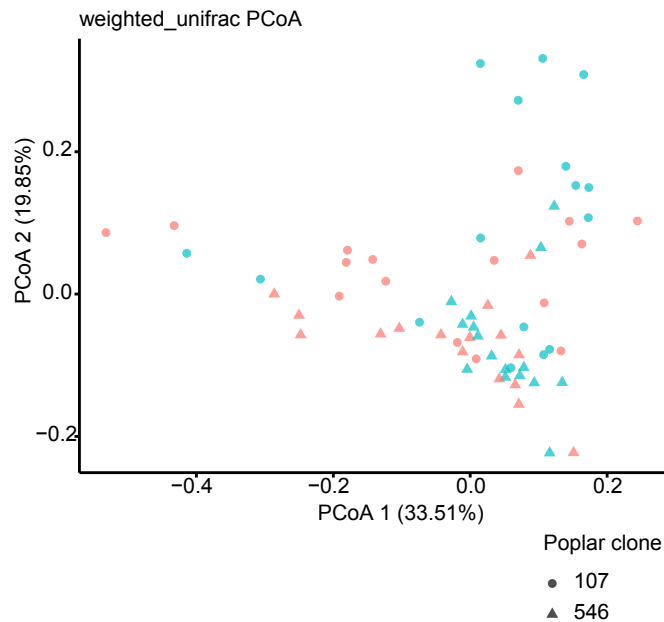

**b** Fungi beta-diversity

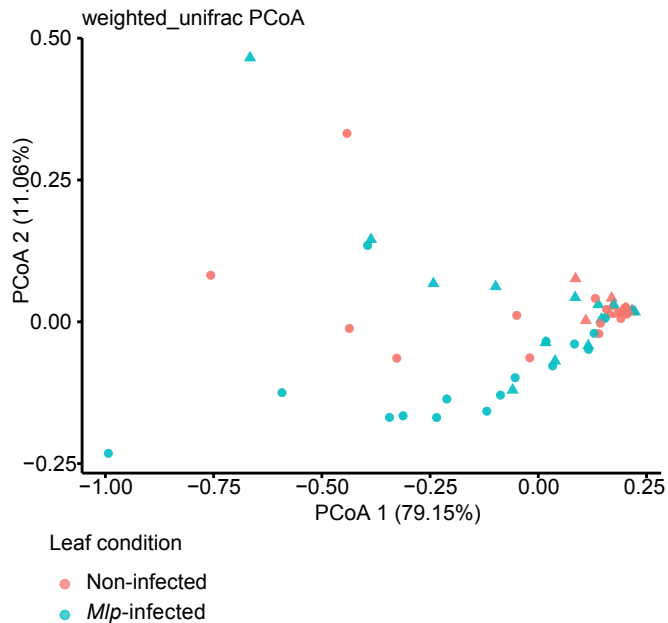

Supplement: Supplementary file 1 [file jof-08-00523-s001.zip › Supplementary files/Figure S4.pdf]
